# The student’s drawing of teacher’s pictorial Value as a predictor of the student–teacher relationship and school adjustment

**DOI:** 10.3389/fpsyg.2022.1006568

**Published:** 2022-10-28

**Authors:** Anna Di Norcia, Anna Silvia Bombi, Giuliana Pinto, Eleonora Cannoni

**Affiliations:** ^1^Department of Social and Developmental Psychology, Sapienza University of Rome, Rome, Italy; ^2^Department of Psychology, University of Florence, Florence, Italy

**Keywords:** teacher authority, student–teacher relationship, school adjustment, primary school, children drawing

## Abstract

This study employs the scale of Value from Pictorial Assessment of Interpersonal Relationships (PAIR) to investigate the links between the importance attributed by primary students to their teachers and two independent measures of scholastic wellbeing, provided by teachers and parents. During middle childhood, the teacher is one of the most significant adults with whom children interact daily; a student–teacher relationship warm and free from excessive dependency and conflict is very important for children wellbeing; however, children’s recognition of teacher importance as an authority figure has been seldom studied. Children aged 7–11 years were individually asked to draw themselves and one of their teachers in two situations (relational Wellbeing and relational Distress); the scale of Value from PAIR was used as a proxy of the importance attributed to teachers in each situation. Teachers completed the Student–Teacher Relationship Scale for Closeness, Conflict, and Dependency of each child; parents answered two items about their children’s School Adjustment. All the study variables were firstly analyzed to check gender and age differences. Boys valorized more than girls the teacher’s figure; however, teachers perceived more Closeness and less Conflict with girls. Dependency and Conflict decreased with age, as well as (albeit slightly) School Adjustment. To assess the links between pictorial valorization of the teacher in Wellbeing and Distress and teachers’ and parents’ evaluations, four separate hierarchical regressions were performed, namely, Closeness, Dependency, Conflict, and School Adjustment, controlling children’s sex and age. The teacher’s pictorial Value in Wellbeing appeared to be related to Closeness and School Adjustment, while a negative relationship emerged between Value and Dependency in Distress. In sum, the recognition of the teacher’s role as an authority figure does not hinder a warm student–teacher relationship and impacts positively on school adjustment. In situations of Distress, dependent pupils showed a diminished appreciation of the teacher’s importance, possibly as a result of a defensive stance.

## Introduction

This study examines the importance and authority attributed by children to their teachers and its links with two independent measures of wellbeing in school: the teacher’s perception of relationship quality and the parents’ perception of school adjustment. As a proxy of children’s consideration for teachers, we use the pictorial valorization of the teacher in two drawings, respectively, of positive and negative interpersonal situations. Drawing is liked by the majority of children and allows them to express ideas, even tacit, without the interference of adults’ conceptions, as it happens in interviews or questionnaires (Freeman and Mathison, 2009); more precisely, the method for collecting and analyzing children’s drawings employed here is Pictorial Assessment of Interpersonal Relationship (PAIR) (Bombi et al., 2007).

### PAIR approach to children’s drawing

The use of drawing to evaluate children’s relationships with significant adults dates back to the application of a projective approach to the representation of family (Hulse, 1951, 1952, cit. in Knoff, 2003), a strategy that was then refined (as reviewed in Handler and Habenicht, 1994; Pace et al., 2021) and also extended to school relationships (Knoff and Prout, 1985). With the projective tradition, PAIR shares the recognition of drawing ecological validity, due to its large practice in children’s life (Kihlstrom, 2021) and its potential for overcoming some limitations of children’s verbal communication, especially about controversial topics (Chandler, 2003). However, PAIR departs from that tradition in some essential aspects.

First, no unconscious mechanism of projection is assumed, but rather a tacit competence to choose images suitable for a communicative goal; in fact, PAIR explicitly requires the child to show, through the drawing, his/her ideas about a specific topic in order to allow the adult to know something about children. This communicative stance is based (1) on the literature on drawing flourished in the 80s of the twentieth century (Thomas and Silk, 1990; Cox, 1992) from which it emerged that even preschoolers are able to adapt their drawings to the researcher’s demands and (2) on a series of empirical studies (summarized in Bombi and Pinto, 1993) demonstrating the children’s capacity to reproduce in recognizable ways spatial arrays, gestures, and features of depicted persons that are emblematic of the relationship and/or the situation to be represented. The children’s choice of relevant information is enhanced by two requests of PAIR: (a) to include oneself in the drawing, which reduces the risk of stereotypic and unrealistic details, and (b) to make two drawings (e.g., “yourself with a friend” and “yourself with a sibling”). This task is manageable even for young children and functions as a conceptual anchor, similar to the semantic differential techniques (Ploder and Eder, 2015); moreover, it is useful for the researcher to keep under control any pictorial idiosyncrasies, not to be interpreted as indicative of ideas on the theme drawn.

PAIR was developed in a historical phase that Gary Ladd (1999) called “the third generation of studies on social competence,” a period characterized by a flourishing of research on the positive side of relationships and on the ability of children to grasp their characteristics. Initially aimed at examining friendship and siblinghood (Bombi and Pinto, 1994; Cannoni, 2002; de Bernart and Pinto, 2005), PAIR has proved equally useful for the representation of a variety of relationships with peers and adults (Bombi and Pinto, 2000) also in intercultural perspective (Pinto et al., 1997; Pinto and Bombi, 2008). In fact, the four main scales constituting PAIR allow the researcher to grasp the fundamental dimensions of human relationships (Fiske, 1992), i.e., the existence of an interpersonal bond tempered by signs of autonomy (scales of Cohesion and Distancing) and partners’ psychological affinity that coexists with disparities of importance (scales of Similarity and Value). For each scale, thanks to construct analysis and empirical studies (detailed in Bombi and Pinto, 1993) adequately informative pictorial elements have been identified and are within the reach of children since the age of 5–6 years.

In sum, PAIR is a research tool designed to avoid some recurrent criticisms leveled against the use of children’s drawing, primarily the need of interpretations heavily dependent on clinical expertise, which are the more controversial requirements of projective methods (Joiner and Schmidt, 1997; Lilienfeld et al., 2000). Moreover, compared with the classic checklists provided for the scoring of projective tests (see a summary in Chandler, 2003), PAIR stands out because it contains analytical criteria to distinguish between intentional and random productions and to evaluate the communicative incidence of details as a function of the increasing complexity of drawings when children become more proficient in their pictorial activity. PAIR has been internationally published by its authors’ research group (Bombi, 2002; Pinto and Bombi, 2008; Lecce et al., 2009; Laghi et al., 2013; Cannoni and Bombi, 2016; Di Norcia et al., 2022a) as well as by other independent researchers (Fraire et al., 2006; Misailidi et al., 2012; Rabaglietti et al., 2012; Sándor et al., 2012; Dimitrova, 2016; Guidotti et al., 2020).

In this study, the scale of Value will be used, which measures the comparative importance of depicted characters according to their reciprocal roles (e.g., adult more valued than child; Bombi and Cannoni, 2001) and relational quality (e.g., enemy less valued than friend; Bombi and Pinto, 1995). The pictorial cues of Value reflect *dominance*, as shown by a figure dimension and upper position, and *personal valorization*, as shown by details provided to its body, clothing, and, if the drawing is not black and white, by the number of colors. Due to these different components, the scale of Value allows the young artists to recognize role disparity as well as personal dignity; for instance, the prominence of parents can be shown by cues of dominance, while the enrichment of the child’s figure moderates the unbalance (Bombi and Cannoni, 2001). Even the realistic constraint of different body sizes between the portrayed characters, which could result in dominance when this is not the case, can be circumvented by the disjunction of the figure size and upper position; this is what second-born children (but not first born!) very often did in a study of siblinghood, e.g., representing themselves standing beside a sitting or crouching brother, and hence as able to “look down” at him (Cannoni, 2002; Lecce and Pinto, 2004). Last but not least, the representation of Value is sensitive to the emotional connotation of the relational circumstances, as shown by the increased disparity between siblings in the case of conflict (Bombi and Pinto, 2000). The scale of Value has been employed in some studies on student–teacher relationship (Bombi and Scittarelli, 1998; Bombi and Pinto, 2000; Fraire et al., 2006) showing that children typically recognize the teachers’ importance, but the possible change of Value in different situations, and the links with other data about the relationship were not examined.

### Teachers’ role between warmth and control

The importance of a harmonious relationship between teacher and primary school children has been demonstrated by many studies, especially thanks to the theoretical and methodological contribution of Pianta (1992) and Pianta and Hamre (2009). According to their studies, and subsequent numerous replications (McGrath and Van Bergen, 2015), a positive student–teacher relationship is characterized by high warmth, low dependency, and low conflict and is associated with students wellbeing (García-Moya, 2020; Zheng, 2022), school adjustment (Bosman et al., 2018), and engagement in learning activities (Pianta et al., 2012; Quin, 2017). Many studies have been devoted to the means of promoting such a relationship thanks to the adoption of positive teaching styles (Kincade et al., 2020; Poling et al., 2022).

Even though it is clear from the above-mentioned studies that teachers have to be proactive in the creation and maintenance of a good relationship with students and that it is their responsibility to act as leaders in the classroom, lesser attention has been paid to children’s recognition of this role. Sociologists and philosophers (Durkheim, 1956; Arendt, 1958) have repeatedly affirmed the importance of authority figures for the transmission of social and cultural heritage. However, as Arendt noted, authority in the absence of a foundation (either theological or political) can reduce itself to the exercise of power and hence be rejected by liberals for the sake of freedom, or accepted by conservatives at the expense of freedom. A teacher has to select school contents and implement learning activities, but this role of “cultural arbiter” (Bourdieu and Passeron, 1990) requires that students freely accept his/her authority.

Adults’ authority over children implies the legitimate use of power in some situations, e.g., in order to prevent a child from doing something that puts him/her in danger. Hence, the use of power cannot be avoided completely in children’s upbringing, and psychologists have tried to trace a path for a just exercise of it, distinguishing between authoritative and authoritarian styles. Baumrind (1966) was the first to test the different outcomes of these styles, which were subsequently conceived as the combination of *demandingness*, which requires the exercise of some power, and *responsivity*, which is the demonstration of acceptance and warmth (Maccoby and Martin, 1983). Studies of parenting showed not only the detrimental effects of excessive power, but also those of its absence. Only a balance of power and acceptance proved to be positive for the child’s wellbeing, ensuring his/her sense of security and at the same time encouraging his/her responsibility and independence.

Recent work has transposed this conceptual framework to the relationship between teachers and students (Turliuc and Marici, 2012). Since the seminal work of Lewin et al. (1939), we know that students’ wellbeing is fostered by a classroom climate in which the teacher is able to use his/her authority without being authoritarian and that if he/she gives up to this role, adopting a *laissez-faire* style, children lose interest in the school activities and behave badly toward each other. Interest in teachers’ authority was recently revived by the fact that students’ unruly behavior and lack of respect constitute for teachers one of the main factors of stress and abandonment of profession (Friedman, 1995; Evans et al., 2019); sometimes even teacher’s victimization has been documented (Kapa et al., 2018). The problem of a correct exercise of authority in class has been examined from a theoretical point of view (Macleod et al., 2012; Lü and Hu, 2021; Chen, 2022) and empirically addressed (Pace and Hemmings, 2007), but research on the correlates of children’s perception of teachers’ authority is still lacking, as far as we know.

Studies of moral development, however, demonstrated that children do understand what authority is and distinguish the spheres of its exercise (Laupa and Turiel, 1986, 1993; Tisak et al., 2000); moreover, Enright et al. (2020) have recently carried out a series of experimental studies on children’s understanding of social status and the associated properties, finding that even 3-year-olds have some idea about the existence of a person “in charge” in some situations and that the role of “boss” implies obedience from subordinates. Overall, these studies suggest that children would apply to teachers their understanding of authority and super-ordinate status.

Is the recognition of authority detrimental to student–teacher relationship? We do not think so. In Italian primary schools, a child-centered style of teaching is prevalent, and the need for resorting to power assertion is not so frequent to disrupt a positive relational style. In such a climate, children’s recognition of the teacher’s status should enhance the teacher’s positive affect.

### Gender and age differences in student–teacher relationship

Closeness and conflict have been demonstrated to be central dimensions of the student–teacher relationship, and they are affected by individual characteristics of the child, including age and gender (Saft and Pianta, 2001). Studies based on teachers’ reports have clearly established that teachers perceive closer and less conflicting relationships with girls than with boys (Baker, 2006; Hajovsky et al., 2017), a result found also in the Italian context (Molinari, 2009) and throughout elementary school (Spilt et al., 2012); studies based on self-reports confirmed more conflict and less closeness for boys (Koomen and Jellesma, 2015). In addition, data on trajectories showed that conflict remained frequent or increased with age only for those students who exhibited high rates of deviant behavior, especially externalizing, which is more common for boys (Lee and Bierman, 2018; Shi and Ettekal, 2021). However, in non-problematic students conflict tended to decrease with age (Wu and Hughes, 2015; Shi and Ettekal, 2021). As regards other age changes, a trend toward a decrease in closeness was generally found from kindergarten to sixth grade (Baker, 2006; Jerome et al., 2008; Spilt et al., 2012). This normative decline of warmth probably reflects a change in classroom organization, more focused on learning goals than on social relationships, as well as a developmental pattern of children growing more independent from adults (Spilt et al., 2012). Lesser attention has been paid to dependency, i.e., a clingy and possessive behavior which can be acceptable in young students, but becomes more and more inappropriate with age. According to a recent meta-analysis (Roorda et al., 2021), dependency is negatively related to various indices of school adaptation and positively related to behavioral difficulties, especially internalizing. In fact, higher autonomy has been found in well-adapted children (Di Norcia et al., 2022b) and developmental trajectories of diminishing dependency have been demonstrated to be beneficial for children’s scholastic wellbeing (Bosman et al., 2018).

### The present study

This study addresses two sets of questions:

(1) To what extent do boys and girls, from second to fifth grade of primary school, recognize the teacher’s greater importance and authority than themselves in different situations of the school life?

(2) How does the degree of importance attributed to the teacher relate to indices of children’s school wellbeing independently provided by teachers and parents?

Based on the literature summarized above, we expect that

• boys and girls alike should acknowledge the importance of the teacher since the early grades of primary school;
• the importance attributed to the teacher should predict a close student–teacher relationship with low conflict and dependency, as well as a positive school adjustment; these outcomes should be also linked to gender and age.

## Materials and methods

### Participants

Participants were 264 students of primary school in a small Italian town: 140 boys and 124 girls, equally distributed in 15 classes from second to fifth grade (ages ranging from 7 to 11 years). The majority of children came from middle-class or lower middle-class families, with 64% of fathers working as employees, 31% self-employed, 2% manager, and 3% unemployed and 50% of the mothers as housewives, 36% employees, and 14% self-employed. Parents’ school degrees were distributed as follows: elementary school (fathers: 4%; mothers: 2%); middle school (fathers: 34%; mothers: 30%); high school (fathers: 44%; mothers: 49%); and college (fathers: 18%; mothers: 18%).

The teachers who took part in this study were women and had a mean age of 46.7 years (range 33–60 years) with an average of about 16 years of service (range 7–30 years). As looping is the typical school policy in Italy, the majority of students had the same classroom teacher throughout the elementary years: Given a 9-month academic year, the time spent together by students and teachers ranged from 6 months (for children who had joined the class in the year of data collection) to 42 months (for children of the fifth grade who had had the same teacher since the first grade).

Informed written consent was obtained from school authorities and teachers. A questionnaire about demographic information was completed by parents, after signing an informed consent ensuring the voluntariness and anonymity of their participation and participation of their children. Children orally accepted informed consent too and completed two drawings. This research and its procedure were approved by the Ethic Committee of Social and Developmental Psychology, Sapienza University.

### Procedure

Our convenience sample was formed on the basis of teachers’ willingness to participate; each of them received the Student–Teacher Relationship Scale (STRS) questionnaire in a sealed envelope with the request to return it to us within a few days. Teachers then helped to reach the students’ families and to distribute and collect the letters of consent and questionnaires.

Drawings were collected by a research assistant. After a short familiarization in the classroom, he brought the children in small groups to another room with tables wide spaced to avoid copying. Here, he explained that each child could show how he/she was getting along with the classroom teacher by drawing him/herself with that teacher in two different moments: “Wellbeing, which is when things go well, you feel fine together, you get along well,” and “Distress, which happens when things are not going well, you do not feel fine together, you do not get along.” Then, each child received two sheets (8 1/2 × 11 in.) entitled: “Myself and my teacher [name of the classroom teacher]—Wellbeing” (WDraw) and “Myself and my teacher [name of the classroom teacher]—Distress” (DDraw). No time limit was set, but to avoid exceeding the 30′ allowed by the school, only paper and pencil drawings were required; all children finished this task within 20′. At the moment of data collection, children were asked to indicate which figure represented the teacher.

### Measures

#### Demographic information schedule

Parents reported the gender (0 = girl; 1 = boy) and age of the son/daughter about whom they were completing the questionnaire and information about their own educational level.

#### Pictorial assessment of interpersonal relationships

Drawings were scored with the above-mentioned scale of Value from PAIR (Bombi et al., 2007) which requires comparing the drawn characters in four subscales: (1) *space occupied*, (2) *dominant position*, (3) *body detail*, and (4) *number of attributes*. In each subscale, the drawn characters receive a zero score if their Value is equal; if their Value is different, a score of 1 or 2 is attributed to the more valued character. Hence, a character can receive 1 or 2 points for each of these qualities: being larger (*subscale 1*), being dominant (*subscale 2*), being more detailed in terms of body parts (*subscale 3*), and being richer in clothing and other accessories (*subscale 4*). As the subscales are independent, each character can receive some points (e.g., character X can receive 1 point for a quite larger size and 2 points for much many body parts and character Y con receive 2 points for a dominant position and 2 points for a very richer clothing); then, the points received by each character can be summed to obtain its individual score of Value (in the example above a score of 3 for character X and a score of 4 for character Y). In alternative, a single score of Value can be obtained focusing on one of the two characters: In this case, the scores attributed to the other (non-focused) character will be first converted in negative points for the focused character and then algebraically summed (in the above example, focusing on character Y, 3 negative scores—corresponding to the value obtained by character X—should be subtracted to its 4 scores, with a final Value score of 1). Following this last strategy, we obtained a single score of Teacher’s Value (TVal) with a possible range from −8 to 8.

Each drawing was rated by two independent judges who had not participated in the data collection and were blind to the aims of the study. The percentages of agreement in each subscale ranged from 83 to 91% for the WDraw and from 80 to 92% for the DDraw. For the final score assignment, they discussed each score on which they disagreed, until a full agreement had been reached.

#### Student–teacher relationship scale

Teachers’ perceptions of the quality of their relationships with individual students were measured using the Student–Teacher Relationship Scale (STRS; Pianta, 1999) in the Italian adaptation for children aged 6–11 years (Molinari and Melotti, 2010). In the Italian instrument, the original dimensions of Conflict and Closeness are strictly replicated. The third original dimension, Dependency, is divided into two components: The first (Dependency) includes also items of conflict and measures a relationship marked by jealousy and relational difficulties and the second (Insecurity) regroups those items that suggest an insecure type of attachment. Finally, three items of Conflict focused on the teacher’s feelings of stress and lack of efficacy, as well as a reversed item of Closeness, give rise to a fifth dimension (Educational Difficulties). All teacher-rated items are based on a five-point Likert-type scale (1 = definitely does not apply to 5 = definitely applies). In this manuscript, we will consider only the scales of Conflict, Closeness, and Dependency, i.e., those more similar to the original instrument. Coefficient alpha reliabilities (α) for Conflict, Closeness, and Dependency scores were 0.79, 0.80, and 0.68, respectively.

#### Parents’ perceived school adjustment

Parents were asked to complete two items (My child’s behavior at school is… and My child’s school performance is…) based on the questionnaire “My child and the school” (Bombi et al., 2014) to evaluate their child’s school adjustment. Response was rated on a four-point scale from poor to excellent. A total score was calculated as a mean of the single score item.

### Data analyses

Data analyses were performed using the statistical program Statistical Package for Social Science (SPSS), version 25.0. Descriptive statistics and bivariate Pearson’s correlations were computed on the study variables. A multivariate analysis of variance (MANOVA) was conducted on the TVal scores in WDraw and DDraw with gender and age as independent variables; ANOVAs were performed on all the other study variables with gender and age as independent variables. Finally, four hierarchical regressions analyses were conducted, in order to investigate the predictors of STRS-Closeness, STRS-Conflict, STRS-Dependency, and School Adjustment among the two scores of TVal in WDraw and DDraw. In the first step, sex and age were entered, and in the second step, TVal scores in WDraw and DDraw were added to the regression equation.

## Results

Descriptive statistics and bivariate Pearson’s correlations are reported in Table 1.

**TABLE 1:** Descriptive statistics and bivariate Pearson’s correlations on study variables.

	1	2	3	4	5	6	7	8	*Range*	*M (SD) boys*	*M (SD) girls*	*M (SD) total*
(1) Gender (1 = boys; 2 = girls)	1								–	–	–	–
(2) Age		1	−0.012	−0.198**	−0.313**	−0.176**	0.022	−0.012	7–11	9.10 (1.21)	9.03 (1.29)	9.07 (1.24)
(3) STRS-closeness			1	−0.276**	0.017	0.012	0.136*	0.055	1–5	3.84 (0.81)	4.14 (0.78)	3.98 (0.81)
(4) STRS-conflict				1	0.494**	−0.063	−0.003	−0.059	1–5	1.38 (0.61)	1.19 (0.47)	1.30 (0.55)
(5) STRS-dependency					1	0.024	−0.086	−0.146*	1–5	1.54 (0.66)	1.60 (0.61)	1.57 (0.64)
(6) School adjustment						1	0.148*	0.000	1–4	3.76 (0.33)	3.80 (0.35)	3.78 (0.34)
(7) Wellness teacher value							1	0.309**	−8 to 8	2.42 (2.43)	1.55 (2.49)	2.01 (2.49)
(8) Distress teacher value								1	−8 to 8	2.36 (3.01)	1.32 (3.68)	1.86 (3.38)

*p < 0.05; **p < 0.01.

Gender differences emerged for the following variables: STRS-Closeness [*F*_(1_,_263)_ = 9.72; *p* = 0.002; η^2^ = 0.04; boys = 3.84 > girls = 4.14]; STRS-Conflict [*F*_(1_,_262)_ = 7.31; *p* = 0.007; η^2^ = 0.03; boys = 1.38 > girls = 1.20]; and TVal [*F*_(1_,_244)_ = 10.09; *p* = 0.002; η^2^ = 0.04; mean of TVal in W and D: boys = 2.39 > girls = 1.44].

A gradual decrease with age was found in STRS-Conflict [*F*_(3_,_263)_ = 8.16; *p* < 0.001; η^2^ = 0.09 second grade = 1.51; third grade = 1.37; fourth grade = 1.15; fifth grade = 1.09] with a significant difference only between third and fourth grades and STRS-Dependency [*F*_(3_,_263)_ = 11.93; *p* < 0.001; η^2^ = 0.12; second grade = 1.78; third grade = 1.69; fourth grade = 1.47; fifth grade = 1.22] with means differing from each grade to the next, except second and third grades. Also, School Adjustment decreased, albeit slowly [*F*_(3_,_263)_ = 2.97; *p* = 0.046; η^2^ = 0.03; second grade = 3.84; third grade = 3.82; fourth grade = 3.72; fifth grade = 3.71] reaching a significant difference only between second and fifth grades.

No interactions between variables were found.

The hierarchical regression analyses conducted to investigate the predictors of student–teacher relationships and school adjustment among the variables measured through children’s drawings showed the following findings. As regards Closeness, step 1 was significant and explained the 0.4% of variance, with female sex predicting a significantly higher closeness and step 2 added a significant increase of *R*^2^ (*p* = 0.03) to the explained variance: Both sex (β = 0.24; *p* < 0.001) and TVal in WDraw (β = 0.16; *p* = 0.01) were significant predictors. For Conflict, only the first step was significant with 0.08% of explained variance, with female sex (β = 0.21; *p* = 0.001) and older age (β = 0.21; *p* = 0.001) predicting lesser Conflict. For Dependency, both step 1 (*R*^2^ = 0.11) and step 2 (*R*^2^ = 0.13) were significant (*p* < 0.001), with younger age (β = −0.32; *p* < 0.001) and lower TVal in DDraw (β = 0.13; *p* = 0.04) predicting more Dependency (see Table 2).

**TABLE 2:** Summary of hierarchical regressions predicting student–teacher relationship from drawing variables.

Step	Predictors	Closeness				Conflict				Dependency			
		*B*	SE B	β	*R* ^2^	*B*	SE B	β	*R* ^2^	*B*	SE B	β	*R* ^2^
1					0.04**				0.08**				0.11**
	Sex (*M* = 1; *F* = 2)	0.33	0.10	0.20**		−0.21	0.07	−0.19**		0.09	0.07	0.07	
	Age	−0.002	0.04	−0.003		−0.09	0.03	−0.21**		−0.15	0.03	−0.32**	
2					0.07*				0.09				0.13*
	Sex (*M* = 1; *F* = 2)	0.38	0.10	0.24**		−0.22	0.07	−0.21**		0.06	0.07	0.05	
	Age	−0.003	0.04	−0.01		−0.09	0.03	−0.21**		−0.15	0.03	−0.32**	
	Wellness teacher value	0.05	0.02	0.16**		−0.001	0.01	−0.01		−0.01	0.02	−0.03	
	Distress teacher value	0.01	0.02	0.04		−0.02	0.01	−0.09		−0.02	0.01	−0.13*	

**p = < 0.01; *p < 0.05.

Finally, as regards the regression on School Adjustment, the first step was significant (*R*^2^ = 0.04) with younger age predicting more adjustment (β = −0.20; *p* = 0.002), the second step was significant explaining the 0.7% of the variance with an increase in a significant *R*^2^ (*p* = 0.03), and Teacher Value in Wellness (β = 0.18; *p* = 0.008) was a significant predictor of school adjustment together with young age (see Table 3).

**TABLE 3:** Summary of hierarchical regressions predicting school adjustment from drawing variables.

Step	Predictors	School adjustment			
		*B*	SE B	β	*R* ^2^
1					0.04*
	Sex (*M* = 1; *F* = 2)	0.02	0.04	0.03	
	Age	−0.05	0.02	−0.20**	
2					0.07**
	Sex (*M* = 1; *F* = 2)	0.04	0.04	0.05	
	Age	−0.05	0.02	−0.20**	
	Wellness teacher value	0.02	0.01	0.18**	
	Distress teacher value	−0.01	0.01	−0.05	

*p < 0.05; **p < 0.01.

## Discussion

This study was aimed at exploring (1) whether and how much primary school children recognize the teacher’s greater importance and authority than themselves in different situations of the school life and (2) whether and how the degree of importance attributed to the teacher relates to indices of children’s school wellbeing independently provided by teachers and parents. The results provided some interesting answers to these research questions, confirming the usefulness of drawing as a way to access children’s perspective on school relationships.

The greater valorization of the teacher with respect to the student indicates a correct perception of the respective roles and is stable from second to fifth grade, without differences between situations of Wellbeing or Distress. The authority of the teacher, of which the pictorial valorization is an index, appears internalized even by the younger participants and is not undermined by the slight drop in enthusiasm as age increases, reported by parents in their assessments of school adaptation. Perhaps this parental evaluation reflects the greater cognitive effort that they perceive in their children as they pass from one class to the next. However, the decreased school adaptation does not imply a deterioration of the relationship with the teacher, which remains close, and indeed less and less conflicting and dependent.

A substantial difference in the representation of Value appears between the adult–child relationship studied here and peer relationships, examined in other studies with PAIR. In a relationship between peers, the relative importance of the partners can be freely negotiated, so that the pictorial Value is affected by relational variants (friendship–enmity; Bombi and Pinto, 1993) and circumstances (harmony–conflict between brothers; Cannoni, 2002; de Bernart and Pinto, 2005). On the contrary, the teacher’s pictorial Value is not undermined by the emotions associated with the pleasant or unpleasant exchanges portrayed, because the teacher’s role is always recognized. One could say that a certain amount of relational Distress is inevitable in the classroom, but this does not disrupt the teacher’s importance when the interactions with students are generally marked by low conflict, low dependency, and high closeness, as it was the case for the participants in this study. Congruent with this reading of the data is also the fact that the importance attributed to the teacher in Wellbeing and Distress does not have significant links with the Conflict in the regression analysis.

**FIGURE 1:**
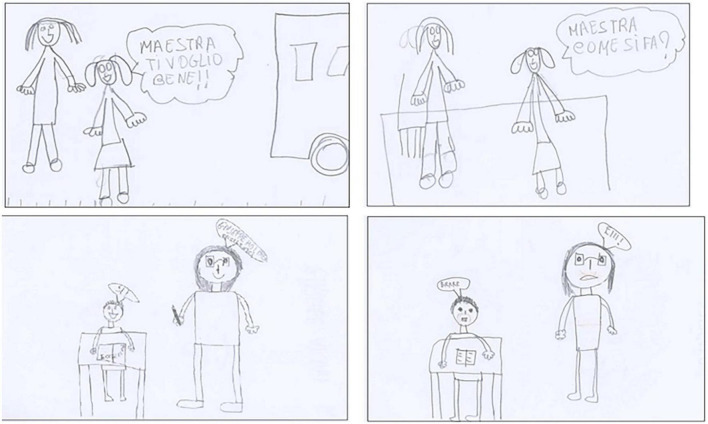
Examples of different valorization of the teacher by gender, independently from the situation. Drawings in the upper section are by the same girl; those in the bottom section are by the same boy; both children are in 2nd grade. Wellbeing drawings are at the left. **(Upper drawing):** Child “Teacher, I love you”. **(Lower drawing):** Teacher “Giuseppe, your work was excellent”; Child “Yes!” Distress drawings are at the right. **(Upper drawing):** Child “Teacher, how should I do it?” **(Lower drawing):** Teacher “Hey!”; Child “Brrr”.

The unexpected gender difference in Value scores is illustrated in Figure 1. Independently from the situations, girls have been less likely to stress the disparity between themselves and the teacher, perhaps because their relationship is closer and less conflicting than that of boys, in line with the cited literature (Baker, 2006; Molinari, 2009; Hajovsky et al., 2017). On the contrary, boys could have developed an image of a powerful person, given the greater frequency with which the teacher must resort to authority to manage their behavior. It is also possible that girls perceive teachers (all women in this study) as akin to themselves; in this direction goes also the fact that, when required to indicate a desired profession as grown-ups, one of the more frequent of girls’ answers is “teaching” (Cavalli, 2014).

The predictive power of Value scores (not found in the case of Conflict) and the usefulness of the dual representation of oneself with a teacher in different circumstances (not evident in the comparisons by age and gender) are instead confirmed by the regression analyses on Closeness, School Adaptation, and Dependency. In fact, the recognition of the teacher’s importance in situations of relational Wellbeing predicts a close relationship and a better school adaptation, while in situation of relational Distress it predicts low dependency. In the first case, the importance of the teacher does not arise from the exercise of power as it happens when she has to correct errors or punish negative behaviors, but is functional mainly to the support given to the student in the school work; the teacher appears as a “significant other” able to use in favor of the student her superiority in terms of knowledge and judgment.

**FIGURE 2:**
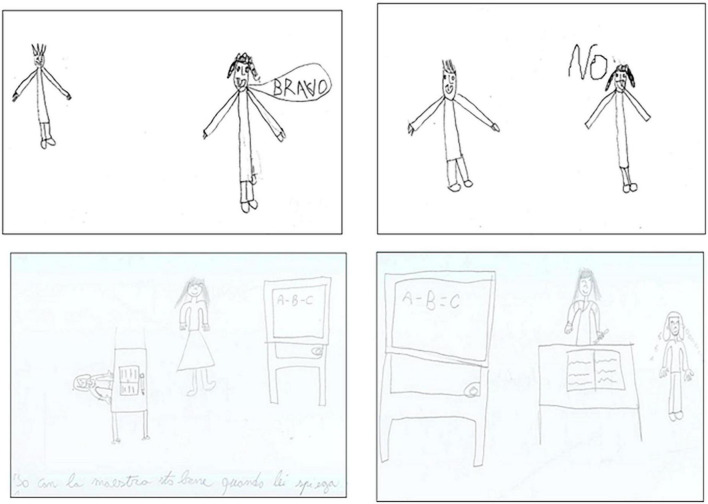
Examples of lower valorization of the teacher in Distress. Drawings in the **(Upper section)** are by the same boy (3rd grade), those in the **(Bottom section)** by the same girl (5th grade): to reduce the teacher’s comparative value the first employed a simple strategy (enhanced self-dimension), while the second used a more complex pictorial plan: she interchanged the respective positions of the figures, showing the teacher’s entire body in the Wellness (caption says: “I feel fine with the teacher when she explains”), and her own entire body in Distress.

Complementary to this result is the negative relationship observed between teacher’s Value and Dependency in Distress; in other words, the ability to recognize the importance and role of the teacher beyond the difficult moment is higher in autonomous children. This result speaks of the importance of uncomfortable moments in the educational context, as a litmus test of progress toward autonomy, which in turn predicts better adjustment to school as shown by the above-quoted literature (Bosman et al., 2018; Roorda et al., 2021; Di Norcia et al., 2022b). The dissonance with the teacher is in fact constitutive of the relationship, as a figure who knows more, who can give rewards or reproaches, and who decides when to work or take a break. The ability to tolerate all of this, even when the teacher does not show herself as a benevolent figure, constitutes a litmus of the student emotional independence. Distress is not well tolerated and translates into an attempt to reduce the teacher’s importance (exemplified in Figure 2) when the child is still at the beginning of the school experience or remains too long in a sort of symbiotic dependency.

Overall, the links highlighted by this research are in line with the most recent literature on the authority of teachers, understood as a necessary component of their role, which can be implemented without compromising the affective quality of the relationship with pupils, and indeed strengthening it (Pace and Hemmings, 2007; Chen, 2022). The focus on students’ perception of authority seems to us a strength of this manuscript, given the scarcity of studies lamented by various authors (Macleod et al., 2012; Lü and Hu, 2021). Another strength is certainly the use of different informants that has made possible to relate the independent evaluations of teachers and parents with the perspectives of children, so as to build a more in-depth picture of the processes taking place in the educational relationship. The use of a solid pictorial tool like PAIR has served to give voice to children since an early age; the drawing proved useful for studying topics, such as teacher’s authority, which are not easy to deal with verbally, especially in reference to problematic interpersonal situations.

We are aware of the study limitations, to begin with the cross-sectional design that does not allow drawing conclusions on the temporal dynamics of the processes examined. A replication in different educational environments, whose specific characteristics should be better known, would be necessary to shed light on the ways in which authority is managed and on its consequences for the students perception; in particular, the effect of gender as a factor able to reduce the disparity of Value should be verified in a sample with male teachers. The collection of drawings outside the school context could also be useful to verify to what extent the teacher is recognized as a significant other when he/she is not present and can be compared to other adult figures (Cameron et al., 2020). Measures of teachers’ ideas on teaching–learning processes (e.g., Vettori et al., 2019; Bessette and Paris, 2020) as well as other measures of pupils’ perspective (e.g., Longobardi et al., 2009; Pezzica et al., 2016) could help to interpret the context in which the teacher’s authority is implemented and pave the way for an examination of individual differences in its perception.

## Data availability statement

The raw data supporting the conclusions of this article will be made available by the authors, without undue reservation.

## Ethics statement

The studies involving human participants were reviewed and approved by the Ethics committee of Developmental and Social Psychology Department, Sapienza University of Rome. Written informed consent to participate in this study was provided by the participants or their legal guardian/next of kin.

## Author contributions

AD performed the statistical analysis and contributed to the conception and design of the study and interpretation. AB wrote the first draft of the manuscript and contributed to the conception and design of the study and interpretation. EC contributed to the data collection, scoring, and interpretation. GP contributed to the conception and design of the study, interpretation, and critical revision of the article. All authors contributed to the manuscript revision, read, and approved the submitted version.
